# Constitutive Expression of Yes-Associated Protein (Yap) in Adult Skeletal Muscle Fibres Induces Muscle Atrophy and Myopathy

**DOI:** 10.1371/journal.pone.0059622

**Published:** 2013-03-27

**Authors:** Robert N. Judson, Stuart R. Gray, Claire Walker, Andrew M. Carroll, Cecile Itzstein, Arimantas Lionikas, Peter S. Zammit, Cosimo De Bari, Henning Wackerhage

**Affiliations:** 1 School of Medical Sciences, University of Aberdeen, Aberdeen, United Kingdom; 2 School of Medicine and Dentistry, University of Aberdeen, Aberdeen, United Kingdom; 3 King’s College London, Randall Division of Cell and Molecular Biophysics, London, United Kingdom; Institut de Myologie, France

## Abstract

The aim of this study was to investigate the function of the Hippo pathway member Yes-associated protein (Yap, gene name Yap1) in skeletal muscle fibres *in vivo*. Specifically we bred an inducible, skeletal muscle fibre-specific knock-in mouse model (MCK-tTA-hYAP1 S127A) to test whether the over expression of constitutively active Yap (hYAP1 S127A) is sufficient to drive muscle hypertrophy or stimulate changes in fibre type composition. Unexpectedly, after 5–7 weeks of constitutive hYAP1 S127A over expression, mice suddenly and rapidly lost 20–25% body weight and suffered from gait impairments and kyphosis. Skeletal muscles atrophied by 34–40% and the muscle fibre cross sectional area decreased by ≈40% when compared to control mice. Histological analysis revealed evidence of skeletal muscle degeneration and regeneration, necrotic fibres and a NADH-TR staining resembling centronuclear myopathy. In agreement with the histology, mRNA expression of markers of regenerative myogenesis (*embryonic myosin heavy chain*, *Myf5*, *myogenin, Pax7*) and muscle protein degradation (*atrogin-1*, *MuRF1*) were significantly elevated in muscles from transgenic mice versus control. No significant changes in fibre type composition were detected using ATPase staining. The phenotype was largely reversible, as a cessation of hYAP1 S127A expression rescued body and muscle weight, restored muscle morphology and prevented further pathological progression. To conclude, high Yap activity in muscle fibres does not induce fibre hypertrophy nor fibre type changes but instead results in a reversible atrophy and deterioration.

## Introduction

Skeletal muscle accounts for ≈38% of body mass in men, and ≈31% in women [1]. Skeletal muscle comprises muscle fibres which are terminally differentiated, multinucleated cells that can be up to 20 cm long in human gracilis and sartorius muscles [2]. Muscle fibres are capable of hypertrophying in response to overload and are able to regenerate following severe injury [3], thanks mainly due to a resident population of stem cells which are termed satellite cells [4]–[6]. Alterations in mature muscle fibre size are determined predominately by a balance in protein synthesis and degradation. Overload-induced muscle hypertrophy is mainly regulated via mTORC1 signalling [7], stimulating protein synthesis, whereas E3 ubiquitin ligases including Atrogin-1 and MuRF-1 regulate protein degradation in catabolic settings. A fast-to-slow fibre phenotype shift and increased mitochondrial biogenesis can also occur in response to stimuli such as endurance exercise training and is mediated by calcineurin-NFAT, AMPK and PGC-1α signalling [8]–[10]. Declines in muscle size and function are key features of both aging and many disease states including muscular dystrophy, cancer and diabetes, and are often major contributors to disability and disease progression [11]. Although progress has been made in uncovering some of the molecular signalling networks responsible for regulating skeletal muscle, the identification of novel pathways that control muscle function still represents a major objective in musculoskeletal research, not least because of the therapeutic potential these pathways may hold.

One such pathway could be the Hippo pathway which is a key regulator of organ growth in mammals [12]. We recently found that all major members of the Hippo pathway are expressed in adult mouse skeletal muscle which suggests a role for Hippo signalling in this tissue [13]. A key member of the Hippo pathway is Yes-associated protein (Yap), a transcriptional co-factor ubiquitously expressed in almost all cell types including skeletal muscle [13]–[15]. Yap activity is negatively regulated by the Hippo pathway via phosphorylation at multiple serine residues including Ser127 by upstream kinase Lats1/2, which retains Yap in the cytoplasm [12], [16], [17]. The protein kinase Mst1/2 interacts with auxiliary proteins ww45 and mob1 to function genetically upstream of Lats1/2, forming the core of the Hippo pathway [12], [16], [17]. Yap can also be regulated independently of the canonical Hippo pathway by proteins including α-catenin [18], β-catenin [19] and angiomotin [20], [21]. Yap, its paralogue Taz (transcriptional coactivator with PDZ-binding motif), and Vgll1-4 (vestigial-like, Vito, Tondu) all bind and co-activate Tead1-4 (TEA/ATTS domain/TEF/scalloped) transcription factors by forming protein complexes via specific Tead-co-factor binding domains [22]–[26]. Co-activated Teads then regulate the expression of target genes by binding via their ≈70 amino acid TEA/ATTS DNA binding domain to so-called MCAT elements (muscle C, A and T; 5′-CATTCC-3′). A chip-on-chip analysis in MCF10A mammary epithelial cells revealed that Yap and Tead1 occupy the same promoters in ≈80% of cases [27], suggesting that Yap co-activation of Tead isoforms is the major mechanism by which Yap regulates gene expression.

The elements and function of the Hippo pathway were first identified during tumour suppressor screens in *drosophila melanogaster*, where knockout of the inhibitory components upstream of the Yap homologue Yorkie resulted in overgrowth [28]. In mammals, subsequent studies have confirmed that the Hippo pathway is a key regulator of organ growth. Conditional liver-specific overexpression of a constitutively active Yap (hYAP1 S127A) induces a ≈4 fold increase in mouse liver size [29], [30]. Expression of a constitutively active Yap or deletion of inhibitory upstream Hippo pathway member ww45/Sav, under cardiomyocyte specific promoters, has been shown to induce an expansion of heart size in developing mice [19], [31], [32]. Similar tissue-expanding functions of Yap have also been described for the skin and intestine [18], [33]–[35]. Yap also acts as an important controller of stem cell identity and fate in several adult stem and/or progenitor populations [36]. Indeed, we have recently demonstrated that Yap promotes satellite cell and myoblast proliferation but inhibits the differentiation of these cells [13], [37].

To date, the function of Yap and Hippo signalling in adult skeletal muscle fibres remains unknown. Given the potent growth promoting properties of Yap in other tissues we hypothesized that Yap could induce muscle fibre hypertrophy *in vivo*. A previous study has demonstrated Tead1, which Yap binds directly, is involved in up regulating α-actin gene expression in hypertrophying chicken muscle [38]. Thus one possibility is that Yap may act via Tead1-4 transcription factors to regulate myofibrillar gene transcription during hypertrophy whilst the mTOR signalling, which is also active after overload, up-regulates translation or protein synthesis. However, more recently it was reported that muscle fibre-specific over expression of Tead1 induces a fast-to-slow fibre type conversion in mice [39]. Thus at this stage it is unclear how Tead1-4 transcription factors, their co-activators and the Hippo pathway regulate the phenotype of skeletal muscle fibres.

Here, we examined the effects of constitutive hYAP1 S127A expression in adult skeletal muscle fibres (but not in satellite cells) [40], [41] using a mouse model in which a tetracycline responsive hYAP1 S127A allele (TRE-hYAP1 S127A) is controlled by a tetracycline transactivator (tTA) driven by the skeletal muscle-specific muscle creatine kinase promoter (MCK-tTA). Unexpectedly, hYAP1 S127A overexpression resulted in muscle degradation, and atrophy, rather than myofibre hypertrophy or any fast-to-slow muscle fibre shift. Specifically, mice lost body weight and suffered from kyphosis and decreased muscle mass after 5–7 weeks of transgene expression. Histological analysis of muscle sections revealed centrally located nuclei, necrotic fibres and nicotinamide adenine dinucleotide-reductionase (NADH-RT) activity resembled that seen in centronuclear myopathy. Interestingly, this degenerative and atrophic phenotype was largely reversible, since removing hYAP1 S127A expression resulted in a regain of body and muscle weight and restoration of a more normal muscle morphology.

## Materials and Methods

### Ethics Statement

All animal procedures were approved by the University of Aberdeen Ethics Review Committee Board and performed under a project license approved by the Home Office under the Animals (Scientific Procedures) Act 1986 (PPL 60/3823).

### Transgenic Mice and Genotyping

MCK-tTA mice were kindly donated by Dr. Kanneboyina Nagaraju, The George Washington University Medical Centre, USA [42]. Transgenic mice carrying the TRE-hYAP1 S127A allele were created and kindly donated by Dr. Thijn R. Brummelkamp, Whitehead Institute for Biochemical Research, USA [34]. MCK-tTA mice were crossed with TRE-hYAP1 S127A mice to generate skeletal muscle specific hYAP1 S127A knock-in mice.

Genotyping was performed on genomic DNA (gDNA) obtained from mouse ear clip samples isolated using a DNeasy Blood and Tissue Kit (Qiagen, USA) following the manufactures instructions. Presence of MCK-tTA and TRE-hYAP1 S127A alleles was then determined using a PCR-gel electrophoresis assays using primers provided in the supporting information (Table S1).

Transgenic mice carrying both alleles (MCK-tTA-hYAP1 S127A) were used as experimental animals and mice carrying a single TRE-hYAP1 S127A allele served as control mice. In this ‘tet-off’ system, transgene expression was suppressed by administration of doxycycline (200 µg/ml) (Sigma) in drinking water supplemented with 2% sucrose, until mice reached 6–10 weeks of age. Activation of transgene expression was then achieved by removal of doxycycline from drinking water.

### Western Blotting

Following dissection, tissue samples were homogenised in RIPA buffer (Tris 50 mM, NaCl 150 mM, SDS 0.1%, Na-Deoxycholate 0.5% and Triton-X 100 1% - supplemented with 50 mM of NaF, 0.5 mM of sodium orthovanadate, 1 mM of EDTA and a protease inhibitor cocktail) at 4°C using a Precellys 24 bead mill homogeniser (Bertin technologies, UK). Samples were centrifugation at 13,000 g for 10 minutes at 4°C and the resultant supernant removed and assayed to determine protein concentration. 50–90 µg of whole protein lysates were separated via SDS-PAGE electrophoresis and transferred to nitrocellulose membranes. Membranes were probed with the following primary antibodies overnight at 4°C: rabbit anti-Yap (a gift from Dr Maris Sudol) rabbit anti-Actin (Sigma), phospho-Akt/PKB Ser473 (Cell Signaling #4060), total Akt/PKB (Cell Signaling #4685), phospho-p70 S6k Thr389 (Cell Signaling #9234), total p70 S6k (Cell Signaling #9202), phospho-Smad2 Ser465/467 (Cell Signaling #3101), total Smad2/3 (Cell Signaling #3102), phospho-Foxo3a Ser253(Cell Signaling #9466), Caspase 3 (Cell Signaling #9665). Primary antibodies were then visualised using species-specific Alexa Fluor 488/555 labelled secondary antibodies (Invitrogen). Bands were quantified using a LiCor Odyssey infrared imager.

### RNA Isolation, cDNA Synthesis and RT-PCR

Following dissection, tissue samples were homogenised in 1 ml TRIzol using a Precellys 24 bead mill homogeniser (Bertin technologies, UK). RNA isolation, cDNA synthesis and end-point PCR was performed as previously described [13] using primers for *hYAP1* and *Gapdh* (primer sequences listed in Table S1). Quantitative RT-PCR was performed on a Roche Lightcycler 480 (Roche, UK) using Taqman assays. qRT-PCR primers and Taqman fluorogenic probes were designed using the Roche Universal Probe Library and can be found in the supporting information (Table S1). All quantification of mRNA was normalised to mouse Gapdh (Applied Biosystems, USA) as an internal control using multiplexing assays. Quantification was corrected for efficiency by use of a standard curve created by the serial dilution of cDNA.

### Skeletal Muscle Histology

Tibialis Anterior (TA) muscles were dissected from mice and then immediately dipped into freezing isopentane (suspended in liquid nitrogen) for 5–10 seconds and stored at −80°C. In a −20°C cryostat-microtome (CM1850UV, Leica, U.K) tissues were cut in a cross sectional orientation through the muscle mid belly using a scalpel then embedded in optimum cutting temperature (OCT) compound (Qiagen, USA) and mounted onto the microtome. Cross sectional cryosections of 5 µm (immunohistochemisty) and 10 µm (basic histology) thickness were then taken from the mid-belly of muscle samples, mounted onto glass slides (Thermo Fisher Scientific, USA) and stored at −20°C.

For haematoxylin and eosin (H+E) staining, frozen TA cryosections were thawed at room temperature, rinsed in distilled H_2_O, incubated in haematoxylin for 3 minutes and then rinsed in tap water. Haematoxylin was then differentiated by dipping slides in 0.3% acid alcohol (1% HCl in 70% ETOH) three times then rinsing in tap water and placing in lithium carbonate for 30 seconds. Following further washes in tap water, slides were finally stained with eosin Y (1% solution) for 1 min. Sections were dehydrated by sequential dipping in 70% and 100% ethanol and then Xylene, before being mounted with coverslips using DPX mounting medium. Measurements of muscle fibre cross sectional area were made manually from images of TA sections (20×magnification) following H+E staining using ImageJ software (NIH – version 1.43, USA). Data was collected from a minimum of 30 fibres per mouse selected at random as previously described [43]. Regenerating muscle fibres were quantified following H+E staining and were expressed as a percentage of total fibres with centrally located nuclei per section (1 section quantified per mouse).

Necrotic muscle fibres were distinguished based on their increased permeability and altered immunogenic properties. Such fibres can be readily identified by incubating muscle sections with fluorescently conjugated-IgG antibodies (secondary antibodies) as fibres undergoing necrosis process a greater affinity to bind IgG’s [44]–[46]. IgG immunostaining was performed as previously described [46]. Briefly, frozen muscle cryosections (5 µm) were thawed and then fixed in 4% PFA for 10 minutes at room temperature in a humidified chamber. Sections were permeabilized in PBS containing 0.2% Triton X-100 for 10 minutes, before being incubated in blocking solution (1% BSA in PBS) for 30 minutes at room temperature in a humidified chamber. Sections were then incubated with an Alexafluor 488-conjugated rabbit anti-mouse antibody (Invitrogen) (1∶200 in PBS) over night at 4°C. The following day, slides were washed twice in PBS and then mounted with Vectashield containing DAPI (Vector Laboratories, USA). Necrotic fibres (IgG+) were quantified by manual counting using ImageJ software (NIH – version 1.43, USA) and displayed as the number of necrotic fibres per section (1 section quantified per mouse).

For Nicotinamide adenine dinucleotide-tetrazolium reductase (NADH-TR) staining, frozen muscle cryosections were thawed and a drop of NADH-TR staining solution (0.68 g Tris base, 2.52 g Tris HCl, 0.10 g Nitro blue tetazolium, dissolved in 100 ml distilled H_2_O –1 mg of NADH added to 1 ml staining solution prior to staining) added to the slides. Samples were then incubated with solution for 30–60 minutes at room temperature in a humidified chamber. Samples were finally washed in tap water before being dehydrated by sequential dipping in 70% and 100% ethanol and then Xylene 1 and Xylene 2 and then mounted with coverslips using DPX mounting medium.

For ATPase staining, TA muscle sections were pre-incubated at pH 10.5 and then processed as previously described [47] in order to distinguish fibre types. Percentages of muscle fibre types were quantified manually by counting stained and unstained fibres from at least three random fields (10×magnification) taken from the core and periphery of muscle sections in line with previously described methods [43].

### Serum Creatine Kinase Assay

Blood samples were collected from mice by cardiac puncture and transferred to EDTA coated mini vacutainer tubes (BD Biosciences, USA) and placed on ice. Following centrifugation for 10 minutes at 3500 rpm, blood plasma (clear layer) was aliquoted into fresh tubes and stored at −20°C. Plasma creatine kinase levels were determined using a CK522 assay kit (Randox Laboratories, UK) following the manufactures instructions. Creatine kinase activity was measured using spectrophotometry at a light absorbance of 340 nm, applying the following formula: Creatine kinase U/l = 4127×Δ A 340 nm/min.

## Results

### Inducible, Skeletal Muscle Specific Expression of hYAP1 S127A *in vivo*

**Figure 1:**
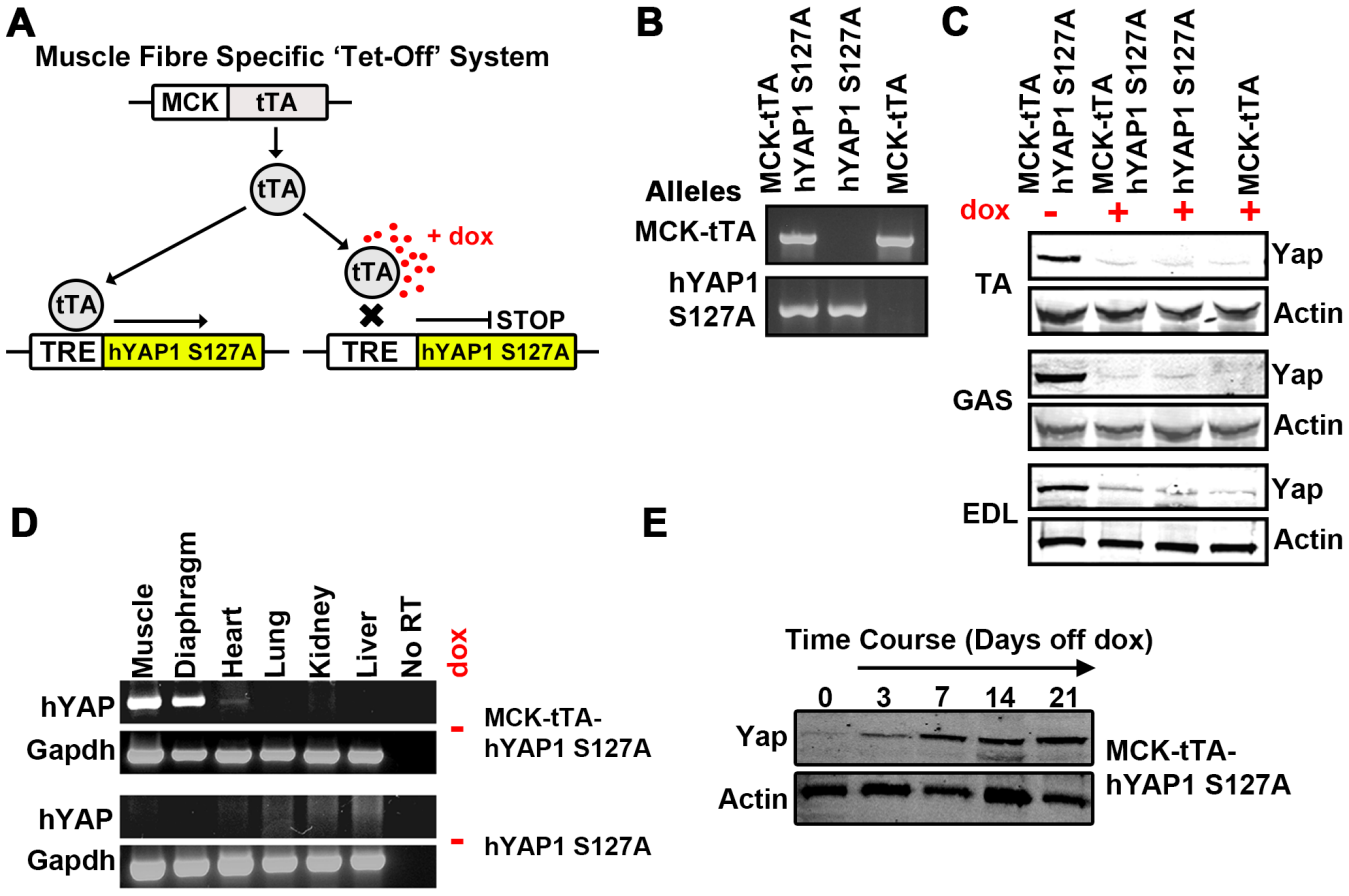
Characterisation of MCK-tTA-hYAP1 S127A mouse model. A) Schematic outline of the inducible ‘Tet-Off’ transgenic system. B) Genotyping of MCK-tTA and TRE-hYAP1 S127A alleles in single and double transgenic mice. C) Yap protein levels in skeletal muscles of transgenic mice of indicated genotypes following 25 days with (+) or without (−) doxycycline (dox). Tibialis anterior (TA), gastrocnemius (Gas) and extensor digitorum longus (EDL). D) Muscle specific expression of hYAP mRNA in MCK-tTA-hYAP1 S127A mice 25 days after doxycycline withdrawal. E) Time course of Yap protein expression in tibialis anterior of MCK-tTA-hYAP1 S127A mice following doxycycline removal.

In order to investigate the function of Yap in adult skeletal muscle fibres, we bred a transgenic knock-in mouse where we could induce the over expression of constitutively active hYAP1 S127A in muscle fibres. For this we crossed mice possessing a tetracycline responsive hYAP1 S127A allele (TRE-hYAP1 S127A) [34] with mice that express the tetracycline transactivator (tTA) driven by the skeletal muscle-specific muscle creatine kinase promoter (MCK-tTA) [42] (Figure 1B). In animals carrying both alleles (MCK-tTA-hYAP1 S127A), this ‘Tet-Off’ system allows skeletal muscle fibre-specific hYAP1 S127A expression to be ‘switched-off’ by doxycycline administration (in drinking water) and ‘switched-on’ by doxycycline withdrawal (Figure 1A). To validate this model, we first bred mice in the presence of doxycycline (restricting *in utero* transgene expression) until offspring were 6–8 weeks of age. Doxycycline was then removed from MCK-tTA-hYAP1 S127A mice for 21 days, which led to an ≈8–12 fold (densitometry, Figure S1) increase in Yap protein levels in all skeletal muscles examined, compared to genotype controls exposed to doxycycline, and single allele controls (Figure 1C). Examination of hYAP1 mRNA in other tissues confirmed that the expression of the transgene was restricted to the muscle linage, especially skeletal muscle but also with a low expression in heart (Figure 1D). No hYAP1 mRNA could be detected in mice carrying a single TRE-hYAP1 S127A allele and were therefore employed as control mice in this study (Figure 1D). Analysing tibialis anterior (TA) muscles following doxycycline withdrawal revealed elevated Yap protein levels from 3 days and peaking at 14–21 days post doxycycline removal (Figure 1E).

### Skeletal Muscle Specific Over-expression of hYAP1 S127A Causes Weight Loss, Kyphosis and Muscle Wasting in MCK-tTA-hYAP1 S127A Mice

**Figure 2:**
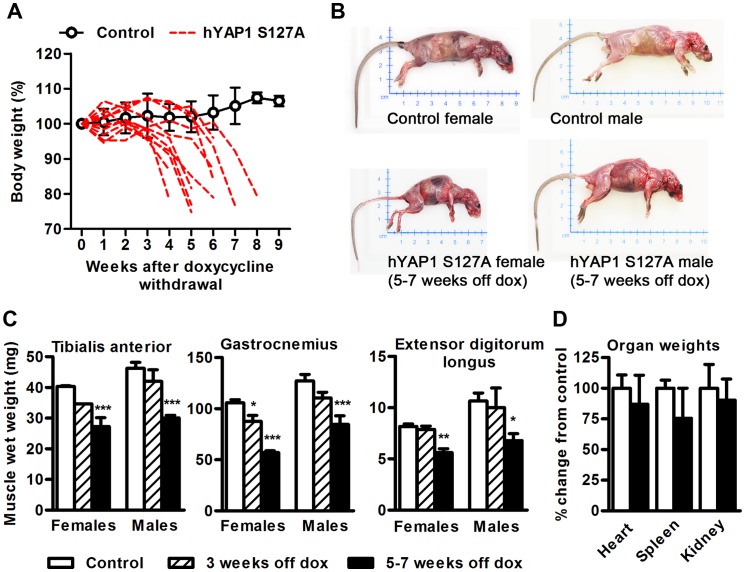
Weight loss, kyphosis and muscle wasting in MCK-tTA-hYAP1 S127A mice following transgene activation. A) Changes in body weight of transgenic mice following doxycycline withdrawal. Dotted red lines indicate the weight of MCK-tTA-hYAP1 S127A mice after doxycycline withdrawal. The black circles represent the mean of mice that remained on doxycycline. B) Gross phenotype of MCK-tTA-hYAP1 S127A versus control mice. C) Skeletal muscle weights of control and transgenic mice following 3 weeks (n = 7) and 5–7 weeks (n = 15) of hYAP1 S127A transgene expression. D) Organ weights taken from control and MCK-tTA-hYAP1 S127A mice (n = 8). All values present mean ±SEM and displayed as percentage change from time point 0 (A) or control (D) or displayed as raw values (C). *p<0.05, **p<0.01, ***p<0.001.

To investigate the phenotype of constitutive hYAP1 S127A over expression in skeletal muscle fibres, doxycycline was withdrawn from the drinking water of 8–10 week old mice to induce transgene expression. The mice were then monitored over 10 weeks for gross changes in phenotype. Induction of hYAP1 S127A over expression appeared to have no effect until 4–6 weeks after doxycycline withdrawal, when mice rapidly lost 20–25% of their body weight compared to controls (Figure 2A). Weight loss was accompanied by an abnormal ‘dragging’ gait of the hind limbs as a result of apparent weakness of the lower limbs (see video S1). An obvious decrease in locomotor activity was also observed as well as hutching, shivering and fur loss, which together indicate declining health. MCK-tTA-hYAP1 S127A mice also developed marked kyphosis (Figure 2B). Eventually transgenic mice reached a stage where they had to be culled based on the experimental protocol and advice by the veterinary doctor. This point was typically reached between 5–7 weeks after doxycycline removal.

Tissue was obtained from mice 3 weeks after removal of doxycycline, when no phenotype was obvious, and from between 5–7 weeks after doxycycline removal, at the point when the mice displayed the most pronounced phenotype (as above). While muscle weight was unaltered after 3 weeks of hYAP1 S127A expression (Figure 2C), TA, gastrocnemius (Gas) and extensor digitorum longus (EDL) muscle weights all significantly decreased (by 33.5%, 39.5% and 33.4% respectfully after 5–7 weeks of transgene expression when compared to control) (Figure 2C). Of note, soleus weight was unchanged (Figure S2). Analysis of other organs from transgenic mice revealed no significant alteration in heart, spleen or kidney weight, although there was a general trend towards a reduction in weight (Figure 2D).

### Over Expression of hYAP1 S127A in Skeletal Muscle Fibres Induces Degeneration, Necrosis, Atrophy and Myopathy

**Figure 3:**
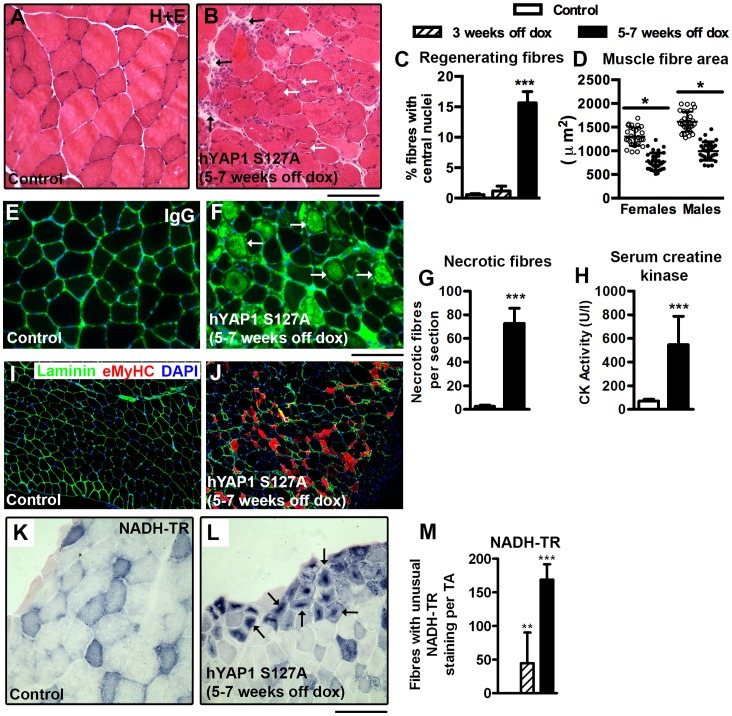
Constitutive expression of Yap induces skeletal muscle degeneration, necrosis, atrophy and features of myopathy. hYAP1 S127A transgene expression was induced by doxycycline (dox) withdrawal for 3 weeks or 5–7 weeks before TA muscles were collected. A & B) H+E staining of cross sections from control and MCK-tTA-hYAP1 S127A mice, revealing central nuclei (white arrows) and infiltrating cells (black arrows). C) Quantification of fibres with centrally located nuclei (n = 18). D) Quantification of muscle fibre cross sectional area (n = 16). E & F) Necrotic fibre staining of cross sections from control and MCK-tTA-hYAP1 S127A mice with fluorescently conjugated IgG. G) Quantification of necrotic fibres, displayed as % of IgG+ myofibres per section (n = 10) H) Serum creatine kinase (CK) activity in control and MCK-tTA-hYAP1 S127A mice (n = 8). I & J) Imunnofluorescent staining of embryonic myosin heavy chain (eMyHC) on cryosections from control and MCK-tTA-hYAP1 S127A mice. K, L & M) Nicotinamide adenine dinucleotide-reductionase (NADH-TR) staining of TA muscle cross sections from control and MCK-tTA-hYAP1 S127A mice (representative image from at least 10 transgenic mice, quantification based on n = 9). All values present mean ±SEM. *P<0.05 ***P<0.001. Scale Bars = 100 µm (H+E, NADH-RT), 100 µm (IgG+eMyHC).

To investigate the basis of the loss of muscle weight, histological analysis of skeletal muscles from MCK-tTA-hYAP1 S127A mice was performed. Haematoxylin-eosin (H+E) staining of TA cross sections revealed myofibres with centrally located nuclei (≈15%) following 5–7 weeks of hYAP1 S127A expression. Central nuclei are a sign of muscle regeneration (white arrows, Figure 3A,B and quantified in Figure 3C). An accumulation of mononucleated cells in the extracellular space between fibres was also prominent in skeletal muscle from transgenic mice (black arrows, Figure 3A,B). Although not specifically characterised, these cells are likely to be immune cells that are recruited to sites of muscle damage in order to clear necrotic debris. This is an immunological response typical for regenerating muscle [48]. Immunostaining for embryonic myosin heavy chain (eMyHC) provided further evidence for the occurrence of ongoing muscle regeneration, with 5–7 weeks of hYAP1 S127A expression inducing the appearance of eMyHC+ fibres in the affected parts of the muscle (Figure 3I, J).

In agreement with these observations, there was evidence of significant muscle fibre necrosis in transgenic mice. Necrotic muscle fibres were distinguished based on their increased permeability and altered immunogenic properties and were identified by incubating muscle cross sections with a fluorescently conjugated antibody [44]–[46]. Muscles from hYAP1 S127A mice displayed large numbers of necrotic fibres (IgG+, white arrows) compared to control mice (Figure 3E,F,G). Five to seven weeks of hYAP1 S127A transgene expression also induced a significant decrease in the cross sectional area of TA muscle fibres (40% mean decrease, Figure 3D), showing that hYAP1 S127A induces muscle fibre atrophy, in addition to degeneration.

Serum creatine kinase (CK) activity is commonly upregulated after skeletal or cardiac muscle damage or in degenerative myopathies [49]. Accordingly, MCK-hYAP1 S127A mice had elevated levels of serum CK activity after 5–7 weeks of transgene expression compared to control mice (Figure 3H).

### hYAP1 S127A Over-expression in Skeletal Muscle Fibres Generates a Phenoytpe with Characteristics of Centronuclear Myopathy

Next, we assessed the metabolic properties of skeletal muscle from control and transgenic mice using nicotinamide adenine dinucleotide-reductionase (NADH-TR) staining as a marker of mitochondrial enzyme activity. Unexpectedly, we found that fibres from the MCK-tTA-hYAP1 S127A mice possessed a strong, concentrated staining patterning in the cores of muscle fibres (Figure 3K, L, quantified in Figure 3M). This adherent mitochondrial distribution resembles the NADH-TR staining of fibres from patients with centronuclear myopathy [50].

### Gene Expression Profile of Muscle Over-expressing hYAP1 S127A is Consistent with Degeneration and Atrophy

To further characterise the skeletal muscle phenotype following induction of hYAP1 S127A, we examined the mRNA expression of several genes involved in regulating skeletal muscle regeneration and atrophy. To this end, TA muscles were dissected from mice at 3 weeks and between 5–7 weeks after doxycycline withdrawal and were compared with TA muscles from adult mdx mice (an animal model of Duchenne muscular dystrophy with a dystrophin knock out mutation) to provide a positive control for a degeneration/regeneration gene expression profile.

**Figure 4:**
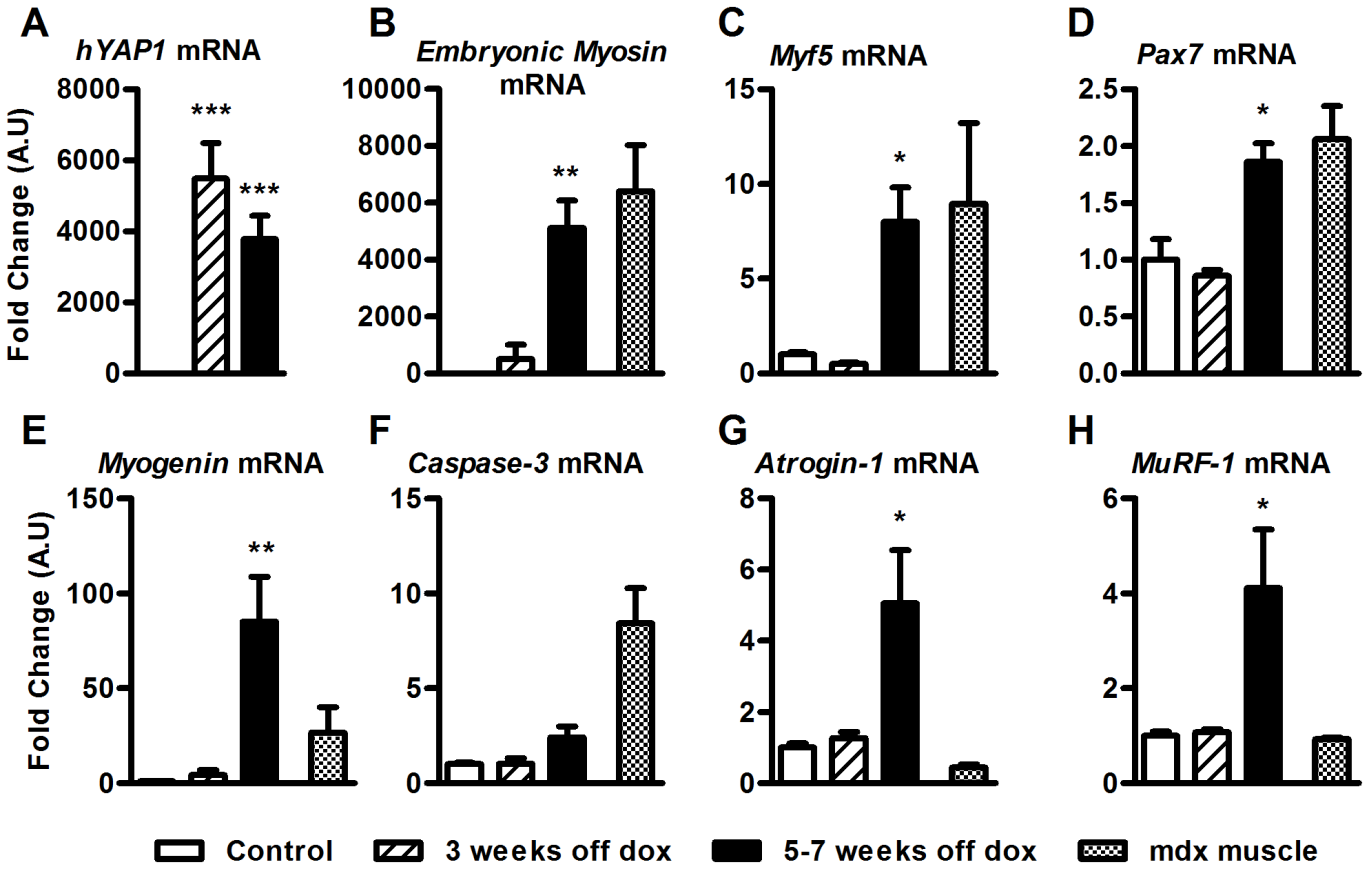
Up regulation in genes associated with muscle regeneration and atrophy in MCK-tTA-hYAP1 S127A mice. TA muscles of transgenic mice were harvested following doxycycline withdrawal at indicated time points and RNA processed for qRT-PCR analysis of A) hYAP1 mRNA, B) embryonic myosin heavy chain, C) Myf5 mRNA, D) Pax7 mRNA, E) myogenin mRNA, F) Caspase-3 mRNA, G) Atrogin-1 mRNA and H) MuRF-1 mRNA. Expression normalised to Gapdh. All values present mean ±SEM (n = 12) and are displayed as fold change relative to control. ***P<0.001, **P<0.01, *P<0.05. Mdx Samples from a mouse model of Duchenne muscular dystrophy.

No differences in gene expression were observed in TA muscles at 3 weeks after doxycycline withdrawal, compared to control mice. However, consistent with our histological observations, TA muscles from MCK-tTA-hYAP1 S127A mice (5–7 weeks off doxycycline) displayed an mRNA expression pattern typically found in regenerating muscle with an up regulation of genes associated with the activation, proliferation and differentiation of satellite cells in response to degeneration/damage. Specifically, *Embryonic myosin heavy chain*, whose expression is restricted to the embryo and postnatal muscle regeneration, was greatly increased (5122 mean-fold increase compared to control, Figure 4B). Similarly, the myogenic regulatory factors *Myf5* and *Myogenin* and the satellite cell marker *Pax7* were also significantly up regulated in muscles from transgenic mice compared to controls (7.9, 85.1 and 2.3 mean-fold increase respectively, Figure 4C, D, E,). These alterations in gene expression were similar to those measured in mdx muscles, providing robust evidence of a degeneration-regeneration skeletal muscle phenotype. However, in contrast to mdx muscles, *Caspase-3* mRNA expression did not increase (Figure 4F). Instead, the mRNA expression of the muscle specific ubiquitin ligases *Atrogin-1* and *Muscle ring finger protein* (*MuRF-1*), which represent key regulators of muscle protein breakdown in various pathological conditions, were significantly increased in muscles from transgenic mice (5–7 weeks off doxycycline) compared to control mice (Figure 4G, H). To test if hYAP1 S127A expression is inducing fibrosis, we also examined the mRNA expression of *Connective tissue growth factor* (*Ctgf*), *Fibronectin* and *Type-1 Collagen (Col1A1). Ctgf* mRNA expression was significantly increased in transgenic mice following 5–7 weeks of doxycycline withdrawal compared to control mice (Figure S3) and there was a trend for an increase in *Col1A1* expression, but this did not reach statistical significance (Figure S3). No significant change in the expression *Fibronectin* was observed (Figure S3). In agreement with these findings, Masson’s trichrome blue staining of skeletal muscle sections from transgenic mice revealed evidence of some limited fibrosis which was not quantified (black arrows, Figure S3).

Since no differences in gene expression were observed after just 3 weeks following doxycycline withdrawal compared to control mice, indicates that alterations in skeletal muscle phenotype appear to take time to manifest following the induction of hYAP1 S127A expression. In addition to the observed reductions in skeletal muscle weight, fibre cross sectional area and evidence of fibre degeneration/necrosis, these biochemical data indicate that hYAP1 S127A expression also correlates with the up regulation of specific molecular pathways associated with muscle protein breakdown and regeneration. However, analysis of selected signalling proteins involved in skeletal muscle protein synthesis and breakdown revealed no significant changes in the phosphorylation status of Akt, p70S6K, Smad2/3 or Foxo3a at activity-related phosphorylation sites (Figure S4), suggesting the Yap appears to be operating via alternative pathways/mechanisms to induce the observed phenotypes. Together these results show that constitutive expression of Yap, unlike observations made in other tissues, appears to drive degeneration and atrophy in skeletal muscle.

### Proportions of Muscle Fibre Types are Unchanged Following Over Expression of hYAP1 S127A *in vivo*

**Figure 5:**
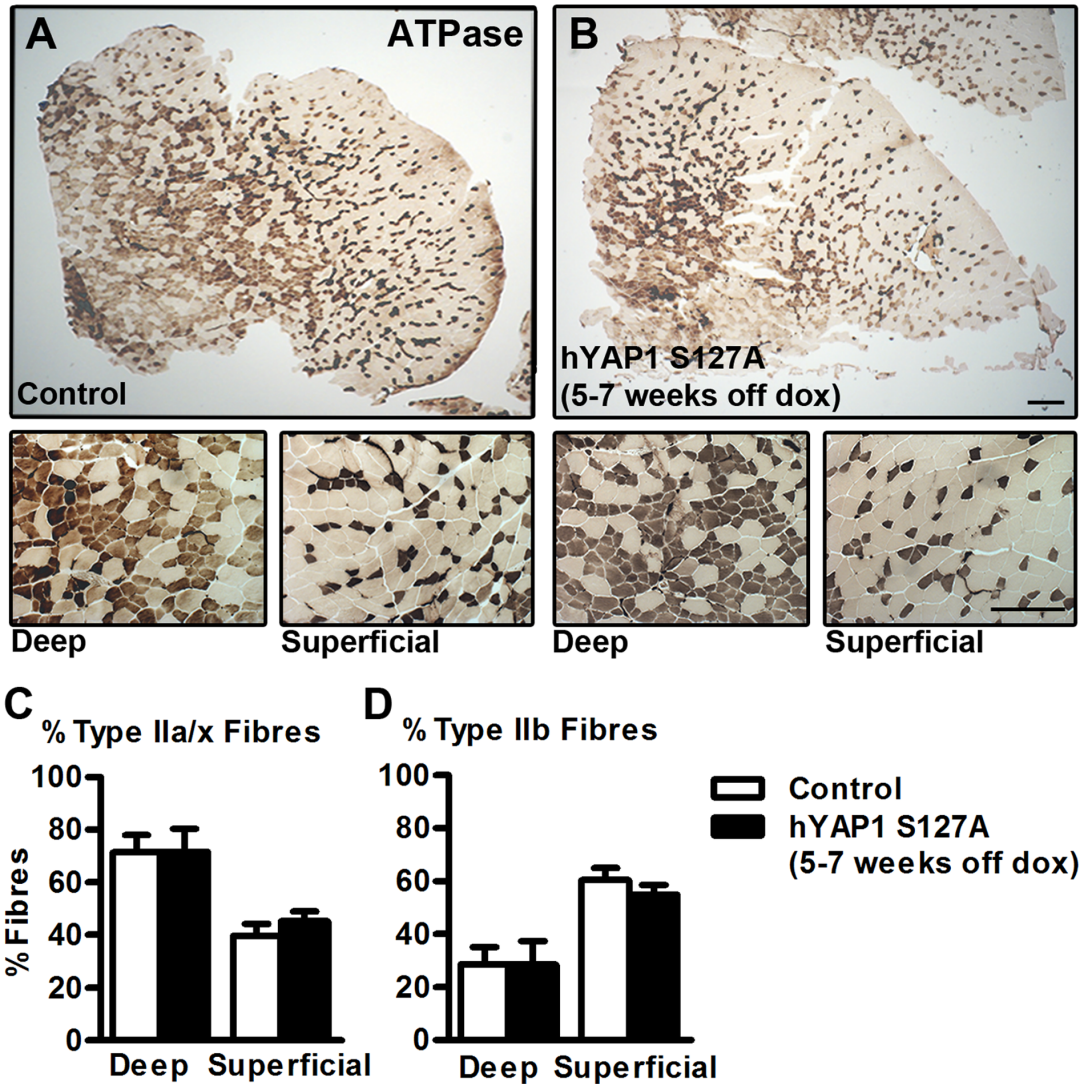
No alteration in muscle fibre type proportions following over expression of hYAP1 S127A in vivo. A+B) hYAP1 S127A transgene expression was induced by doxycycline (dox) withdrawal for 5–7 weeks before TA muscles were collected, sectioned and subjected to ATPase histochemical staining following a pH10.5 pre-incubation. C+D) Quantification of the proportion of Type IIa/x (dark) and Type IIb (light/no stain) fibres in the deep and superficial portions of muscle sections. Values present mean ±SEM (n = 10) and displayed as percentage of total fibres per image from 3 representative images per mouse. Scale Bar = 200 µm.

Given that Yap is known to interact with Tead transcription factors [27], [37] which are involved in the regulation of muscle fibre type, we examined if MCK-tTA-hYAP1 S127A mice displayed an alteration in the proportion of Fast Type IIa/x/b fibres compared to control mice. TA cross sections from transgenic mice 5–7 weeks after doxycycline withdrawal were subjected to ATPase histochemical staining. As the TA is comprised almost exclusively of type IIa/x/b fibres, sections were pre-incubated at pH 10.5 followed by incubation with ATP and visualisation of the Pi generated by the reaction, allowing distinction between Type IIa/x (dark stained) and Type IIb (light/no stain) fibre types as described in [47] (Figure 5A, B). Quantification from representative images taken from the deep and superficial portion of TA muscle cross sections revealed no significant difference in Type IIb versus IIa/IIx fibre type proportions between MCK-tTA-hYAP1 S127A and control mice (Figure 5C, D).

### Muscle Atrophy can be Rescued by Reversal of hYAP1 S127A Over-expression *in vivo*

**Figure 6:**
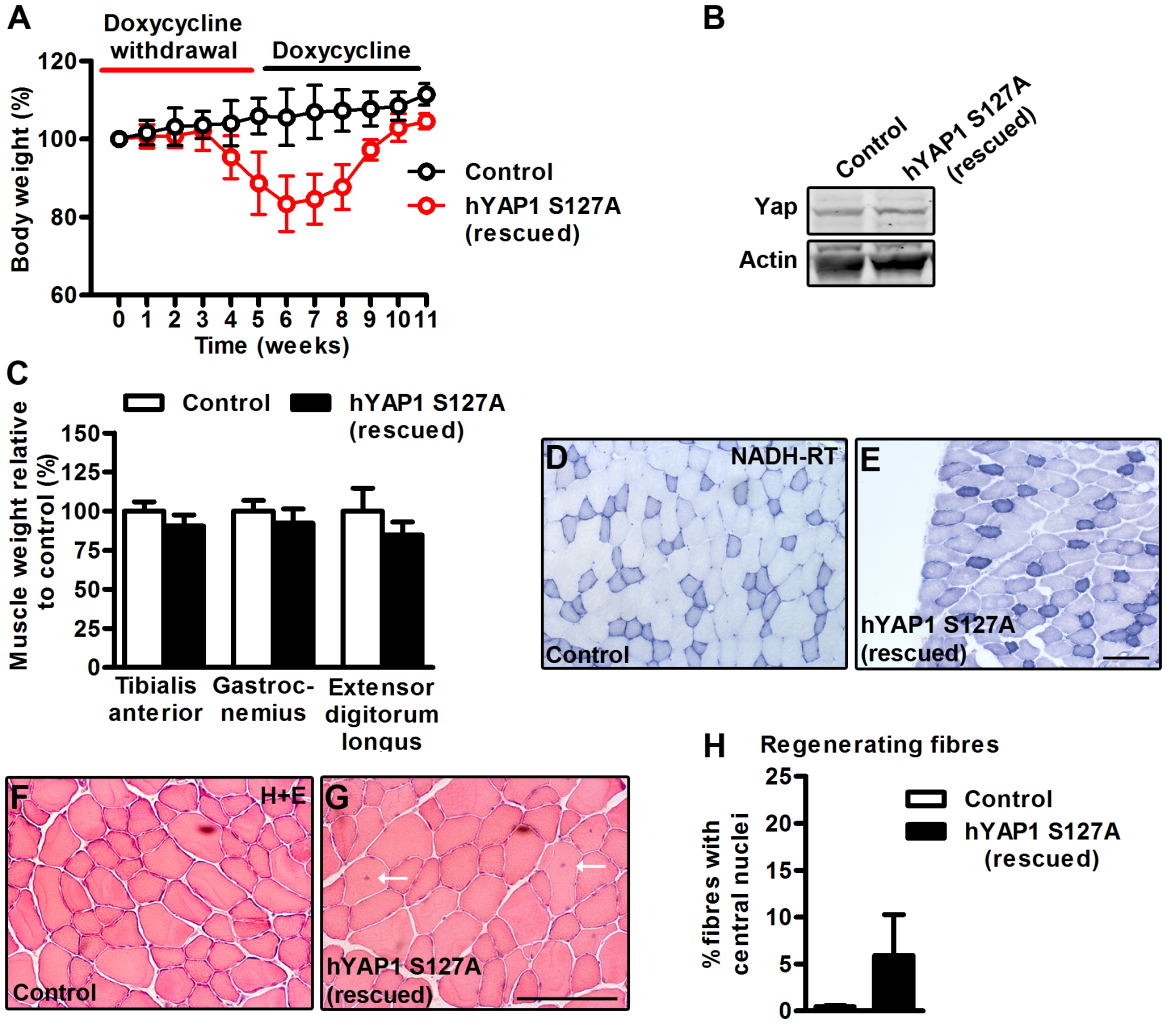
Re-administration of doxycycline partially rescues muscle phenotype induced by hYAP1 S127A expression. hYAP1 S127A transgene expression was induced for 5 weeks and then stopped by the re-administration of doxycycline (dox) for 5 weeks (hYAP1 S127A rescued). A) Weekly changes in mouse body weight throughout trial B) Western blot of Yap protein expression from control and hYAP1 S127A rescued mice. C) Quantification of TA, GAS, EDL muscle weights from control and hYAP1 S127A rescued mice. D+E) Representative images of NADH-TR stained TA cross sections of control and hYAP1 S127A rescued mice. F+G) Representative images of H+E staining from TA cross sections of control and hYAP1 S127A rescued mice. E) Quantification of centrally located nuclei. F) Western blot of Yap protein expression from control and hYAP1 S127A rescued mice. Values present mean ±SEM (n = 6). Weights are displayed as percentage change from control. Central nuclei are displayed as the percentage of muscle fibres that had central nuclei. Scale bars = 100 µm.

To test whether the skeletal muscle degeneration and wasting in MCK-tTA-hYAP1 S127A transgenic mice is dependent on sustained expression of hYAP1 S127A, we conducted a rescue experiment. For this, doxycycline was first withdrawn from the drinking water of 8–10 week old mice, inducing weight loss as previously observed (Figure 6A). Doxycycline was then re-administered after 5 weeks to stop hYAP1 S127A over-expression. Re-adminstration of doxycycline led to a gradual recovery of mouse body weight (Figure 6A) and normalisation of Yap protein levels (Figure 6B) after 5 weeks. Examination of skeletal muscles 5 weeks after re-administration of doxycycline revealed an almost complete restoration of muscle weight (Figure 6C) and partial recovery of histology (Figure 6D,E,F,G). NADH-RT staining resumed an appearance comparable to control mice (Figure 6D,E). Muscle fibres with centrally located nuclei were still present (≈5% of total fibres), (white arrows Figure 6D, quantified Figure 6E). These data clearly indicate that inactivation of hYAP1 S127A expression is sufficient to prevent further pathological progression as well as rescue aspects of the muscle phenotype that was induced by hYAP1 S127A over expression.

## Discussion

Previous observations with YAP in other tissues and organs suggested that constitutive expression in muscle may induce myofibre hypertrophy or alter muscle fibre type composition. Using an inducible, tissue specific hYAP1 S127A knock-in mouse however, we have found that instead, constitutive expression of hYAP1 S127A in adult muscle fibres caused muscle atrophy and degeneration. Importantly, these changes could be largely reversed by then reversing the hYAP1 S127A over-expression.

Five to seven weeks of hYAP1 S127A expression induced a significant decrease in TA, GAS and EDL weights, but no change in soleus weight,. The reason for this muscle group specificity is unclear, although not uncommon in muscle wasting phenotypes. Selective sparing of the soleus has been observed in both human and rodent models of cachexia and there is evidence that the high proportion of type I fibres may be protective against catabolic stimuli [51], [52]. Analysis of skeletal muscle fibre cross sectional area from induced hYAP1 S127A knock-in transgenic mice revealed muscle weight loss due to muscle fibre atrophy. In agreement with this observation, mRNA expression of the muscle specific E-3 ubiquitin ligases, MuRF1 and Atrogin-1 were significantly up-regulated following 5–7 weeks of hYAP1 S127A expression, but not after 3 weeks of doxycycline withdrawal nor in *mdx* mice. MuRF-1 and Atrogin-1 are up-regulated during muscle atrophy and target muscle-specific proteins for degradation by the proteasome [53]. There are many similarities between MCK-tTA-hYAP1 S127A mice and MuRF-1 knock-in mice [54]. Although E-3 ubiquitin ligases have been shown to negatively regulate various members of the Hippo pathway [55], including Yap [56], whether Yap is capable of directly driving MuRF-1 or Atrogin-1 expression, or is involved in their protein regulation, remains unknown. There is currently no evidence linking Yap to the up regulation of ubiquitin or proteasome pathways. Recent evidence in breast cancer cells has shown Yap is instead capable of protecting specific proteins against E-3 ubiquitin ligase mediated polyubiquitination [57]. MuRF-1 and Atrogin-1 mRNA levels were not up-regulated after 3 weeks of hYAP1 S127A expression, suggesting that Yap is unlikely to be targeting these genes directly in adult skeletal muscle fibres.

In addition to muscle atrophy, hYAP1 S127A over-expression also led to muscle degeneration. Histological analysis of skeletal muscle sections from transgenic mice revealed evidence of centrally-located nuclei and the appearance of embryonic myosin heavy chain-expressing fibres: hallmarks of muscle regeneration. In support of these observations, molecular markers of regenerative myogenesis including *embryonic myosin heavy chain*, *Pax7*, *Myf5* and *Myogenin* were up-regulated in a genetic signature indicative of satellite cell activation and differentiation during muscle repair [6], and consistent with the gene expression profile of *mdx* muscle. We have recently shown that Yap influences the expression of the myogenic regulatory factors Myf5 and Myogenin as well as Pax7 in myoblasts [13], [37]. Importantly, the *MCK* promoter is active in differentiated muscle fibres [40], [41], but not in satellite cells or other myogenic stem cells or progenitors until they undergo myogenic differentation. No change in *Pax7*, *Myf5* or *Myogenin* mRNA was observed 3 weeks following hYAP1 S127A induction, indicating that such gene expression changes are likely the result of muscle stem cell-mediated regeneration occurring within the muscle tissue of transgenic mice, rather that hYAP1 S127A directly targeting these genes in muscle fibres.

Previous studies in mouse and *Drosophila melanogaster* have highlighted Yap and Yorkie (*Drosophila melanogaster* homologue of Yap) as potent promoters of cell growth, survival and resistance to death in almost all cell and tissue types investigated. Yet, in adult skeletal muscle fibres, constitutive expression of Yap drives atrophy, degeneration and necrosis. These data highlight important tissue specific differences in the function of Yap and suggest that Yap does not always represent a *universal* growth regulator as has been suggested [30]. Interestingly, this study provides one of the first investigations into the function of Yap in mammalian, post-mitotic cells *in vivo*. Previous studies have focused on cell populations within the liver (oval cells), intestine (crypt progenitors), skin (epidermal progenitors) and embryonic heart (cardiomyocytes) that can all still proliferate [18], [30]–[35]. In these cell types Yap is able to induce growth by driving cell cycle and increasing cell number (hyperplasia). However, while mouse muscle initially grows postnatally by both hypertrophy (increase in myofibre size) and an increase in myonuclear content from satellite cells, after 3 weeks of age any expansion in muscle size occurs almost exclusively through hypertrophy [58]. This implies that Yap is unable to promote growth without cell division, in the same manner as it does in other tissues. Indeed, a recent study investigating Yap gain of function in foetal mouse hearts provided robust evidence for the growth promoting function of Yap coming entirely from enhanced proliferation, rather than a hypertrophy of cardiomyocytes [31]. These cell type specific differences in proliferative capacity may go some way in explaining the distinct effect that Yap over expression has in skeletal muscle when compared to other tissues.

No significant change in the mRNA expression of *Col1A1* and *Fibronectin* was observed in skeletal muscles of transgenic mice, suggesting 5–7 weeks of hYAP1 S127A over expression do not cause fibrosis. However, hYAP1 S127A did induce a significant increase in *Ctgf* mRNA. The *Ctgf* gene has three MCAT elements in its proximal promoter (MCAT) and generally responds to hYAP1 S127A over expression [27]. A recent study has shown over expression of Ctgf in skeletal muscle *in vivo* induces a similar phenotype as that seen in MCK-tTA-hYAP1 S127A mice [59]. Morales *et al.,* observed adenoviral delivery of *Ctgf* in mouse TA muscle induced myofibre necrosis, damage and degeneration within days of transgene induction [59]. Assessments of muscle function also showed over expression of *Ctgf* produced a significant reduction in isometric contraction force. Although *Ctgf* is an established transcriptional target of Yap [27] and its expression is markedly increased in skeletal muscles of MCK-tTA-hYAP1 S127A mice, it remains to be seen if this may represent a possible mechanism of action responsible for the muscle phenotypes observed.

Histochemical ATPase staining revealed no significant changes in the percentage of Type IIa/x and Type IIb fibres in TA muscle, suggesting that hYAP1 S127A transgene expression does not induce a shift of fibre type. In mouse, skeletal muscle specific over expression of Tead1, a known DNA binding partner of Yap, has been shown to induce an increase in the abundance of Type IIa myosin heavy chain protein and decrease in Type IIb myosin heavy chain isoform expression, indicative of a shift to a ‘slower’ contractile phenotype [39]. These results are not consistent with the phenotype of MCK-tTA-hYAP1 S127A mice following transgene activation allowing speculation that Yap is unlikely to be regulating the same target genes as the Tead1 knock-in mouse model [39] and is therefore operating through an alternative mechanism. Further analysis is required to confirm this.

An unexpected histological feature of skeletal muscles from MCK-tTA-hYAP1 S127A mice was the appearance of groups of myofibres with intense, centrally-located NADH-TR staining. Interestingly, similar NADH-TR staining patterns are observed in some myopathies [50], [60]–[62]. For example, centronuclear myopathies, a group of rare human congential myopathies, are defined pathologically by the presence of centrally-located nuclei [62], and share comparable metabolic staining features as when hYAP1 S127A is over-expressed. Skeletal muscle characteristics of centronuclear myopathy include increased oxidative enzyme activity and glycogen staining in the central areas of muscle fibres [50]. Sections taken from patient muscle biopsies stained for oxidative enzyme activity (NADH-TR/NBT) show fibres with a pale halo at the periphery of the fibre, with central areas of fibres occupied by aggregates of mitochondria and glycogen particles [50], [60]. Such metabolic staining patterns appear to closely match those observed in groups of muscle fibres from MCK-tTA-hYAP1 S127A mice following transgene activation. In humans, the genetic causes of centronuclear myopathies are heterogeneous. Genetic linkage studies have found X-linked recessive centronuclear myopathy is caused by mutations in the gene *MTM1* (encodes protein Myotubularin) [63], whereas autosomal dominant and recessive forms are caused by mutations in *DNM2 (*encodes protein Dynamin 2) [64] and *BIN1* (encodes protein Amphiphysin 2) [65] genes respectively. There is currently little direct or indirect evidence linking Yap with centronuclear myopathy or any of the genes associated with the disease, with no reports demonstrating disruption of Hippo signalling or Yap in any myopathies to date. Whether the patterns of metabolic staining in skeletal muscles of MCK-tTA-hYAP1 S127A mice are merely analogous to CNM or provide evidence for an involvement of Yap in pathology of centronuclear myopathy is unknown.

In summary, we have shown that unlike other tissues, including liver, skin and heart, constitutive expression of Yap in skeletal muscle fails to induce growth. Instead, we found that hYAP1 S127A expression in adult muscle fibres induces muscle atrophy, degeneration and features of myopathy, providing important insight into the tissue specific phenotypes induced by Yap over-expression. This is reversible though, since extinguishing hYAP1 S127A expression leads to a rapid restoration of muscle weight and morphology. Based on our findings, sustained expression of Yap is unlikely to provide a viable target for future therapies in the treatment of muscle wasting conditions.

## Supporting Information

Figure S1 **Quantification of Yap protein levels from MCK-tTA-hYAP1 S127A mice following doxycycline withdrawal.** Densitometry of Western Blots in Figure 1C, showing Yap protein levels relative to actin in skeletal muscles of transgenic mice of indicated genotypes following 25 days with (+) or without (−) doxycycline (dox). All values present mean ±SD (n = 3) and are displayed as fold change relative to control (mice carrying single hYAP1 S127A allele) ***P<0.001.
(TIF)

Figure S2 **Weights of soleus muscles from MCK-tTA-hYAP1 S127A and control mice.** hYAP1 S127A transgene expression was induced by doxycycline (dox) withdrawal for 5–7 weeks and then soleus muscles were collected and weighed. All values present mean ±SEM and displayed raw values (n = 15).
(TIF)

Figure S3 **No significant fibrotic tissue accumulation in skeletal muscles of MCK-tTA-hYAP1 S127A mice.** TA muscles of transgenic mice were harvested 5–7 weeks following doxycycline (dox) withdrawal. RNA was isolated and processed for qRT-PCR analysis of A) *Contective tissue growth factor* (*Ctgf*) mRNA, B) *Fibronectin* mRNA C) *Type-1 Collagen (Col1A1)* mRNA. D+E) Masson’s trichrome blue staining of TA sections from control and MCK-tTA-hYAP1 S127A mice. Black arrows highlight small areas of fibrotic tissue (blue staining). mRNA expression normalised to *Gapdh* mRNA. Values present mean ±SEM and displayed as fold change relative to control mice (n = 8). ***P<0.001.
(TIF)

Figure S4 **Cell signalling in skeletal muscle of MCK-tTA-hYAP1 S127A.** TA muscles of transgenic mice were harvested 3 weeks or 5–7 weeks following doxycycline withdrawal. Protein was isolated from muscle samples and processed for Western blotting. Membranes were probed with indicated antibodies. Densitometry was performed and normalised to indicated total proteins for A) pAkt, B) p-p70S6K, C) pSmad, D) pFoxo3 and E) caspase. Values present mean ±SEM and displayed as fold change relative to control mice (n = 4–8).
(TIF)

Table S1 **Primer details.** PCR primers for genotyping, end-point PCR primers for *hYAP* and *Gapdh* mRNA and qRT-PCR primers and probes (Roche Universal Probe Library).
(DOCX)

Video S1 **Videos of MCK-tTA-hYAP1 S127A (hYAP1 S127A) and control mice following 5–7 weeks of doxycycline withdrawal.** Videos highlight decreased locomotion, hutching and gait impairment seen in transgenic animals.
(WMV)
