# Supplementary figures and images for: Constitutive Expression of Yes-Associated Protein (Yap) in Adult Skeletal Muscle Fibres Induces Muscle Atrophy and Myopathy
Source: PLoS One. 2013 Mar 27;8(3):e59622. doi: 10.1371/journal.pone.0059622 (PMC3609830; doi:10.1371/journal.pone.0059622)

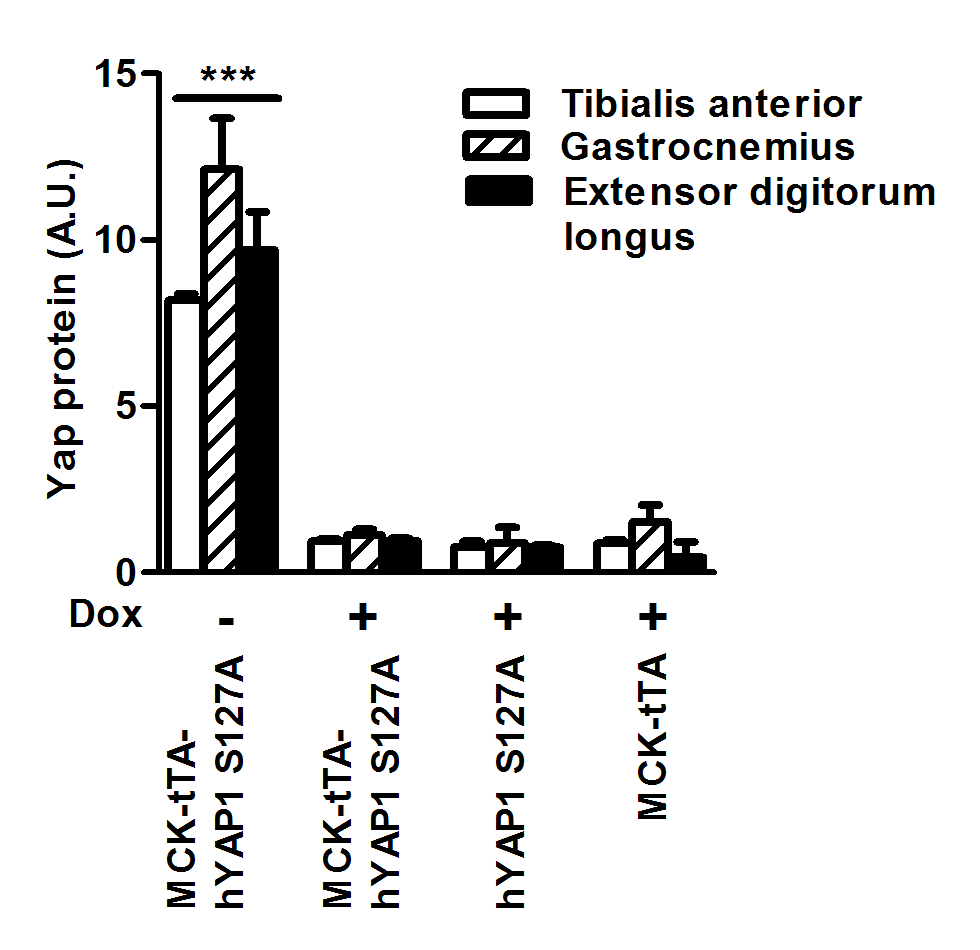

Supplement: Figure S1 — Quantification of Yap protein levels from MCK-tTA-hYAP1 S127A mice following doxycycline withdrawal. Densitometry of Western Blots in Figure 1C, showing Yap protein levels relative to actin in skeletal muscles of transgenic mice of indicated genotypes following 25 days with (+) or without (−) doxycycline (dox). All values present mean ±SD (n = 3) and are displayed as fold change relative to control (mice carrying single hYAP1 S127A allele) ***P<0.001. (TIF) [file pone.0059622.s001.tif]

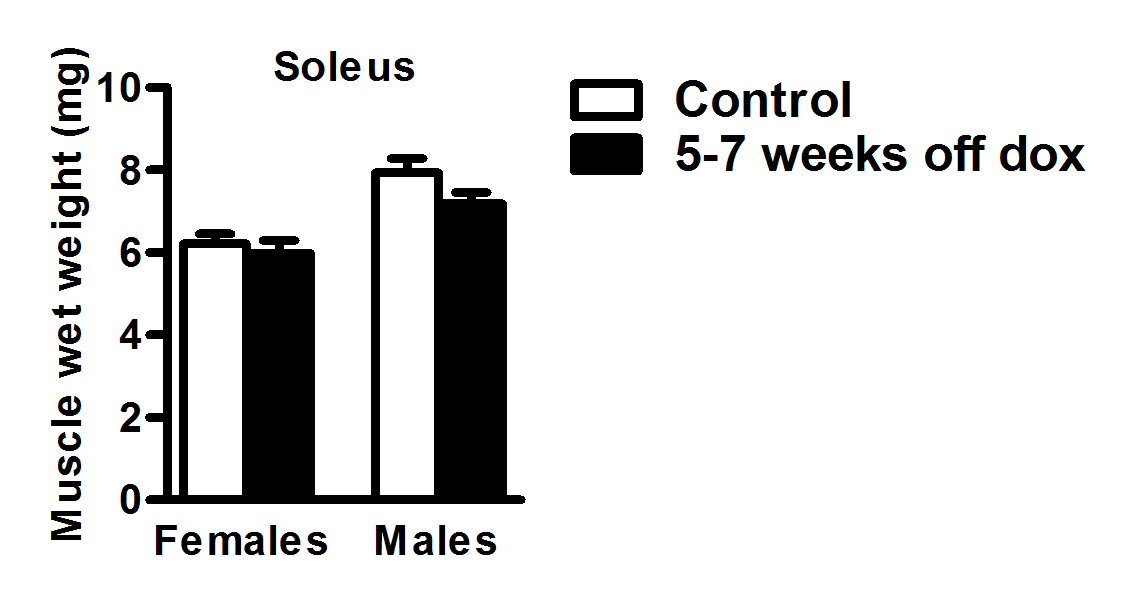

Supplement: Figure S2 — Weights of soleus muscles from MCK-tTA-hYAP1 S127A and control mice. hYAP1 S127A transgene expression was induced by doxycycline (dox) withdrawal for 5–7 weeks and then soleus muscles were collected and weighed. All values present mean ±SEM and displayed raw values (n = 15). (TIF) [file pone.0059622.s002.tif]

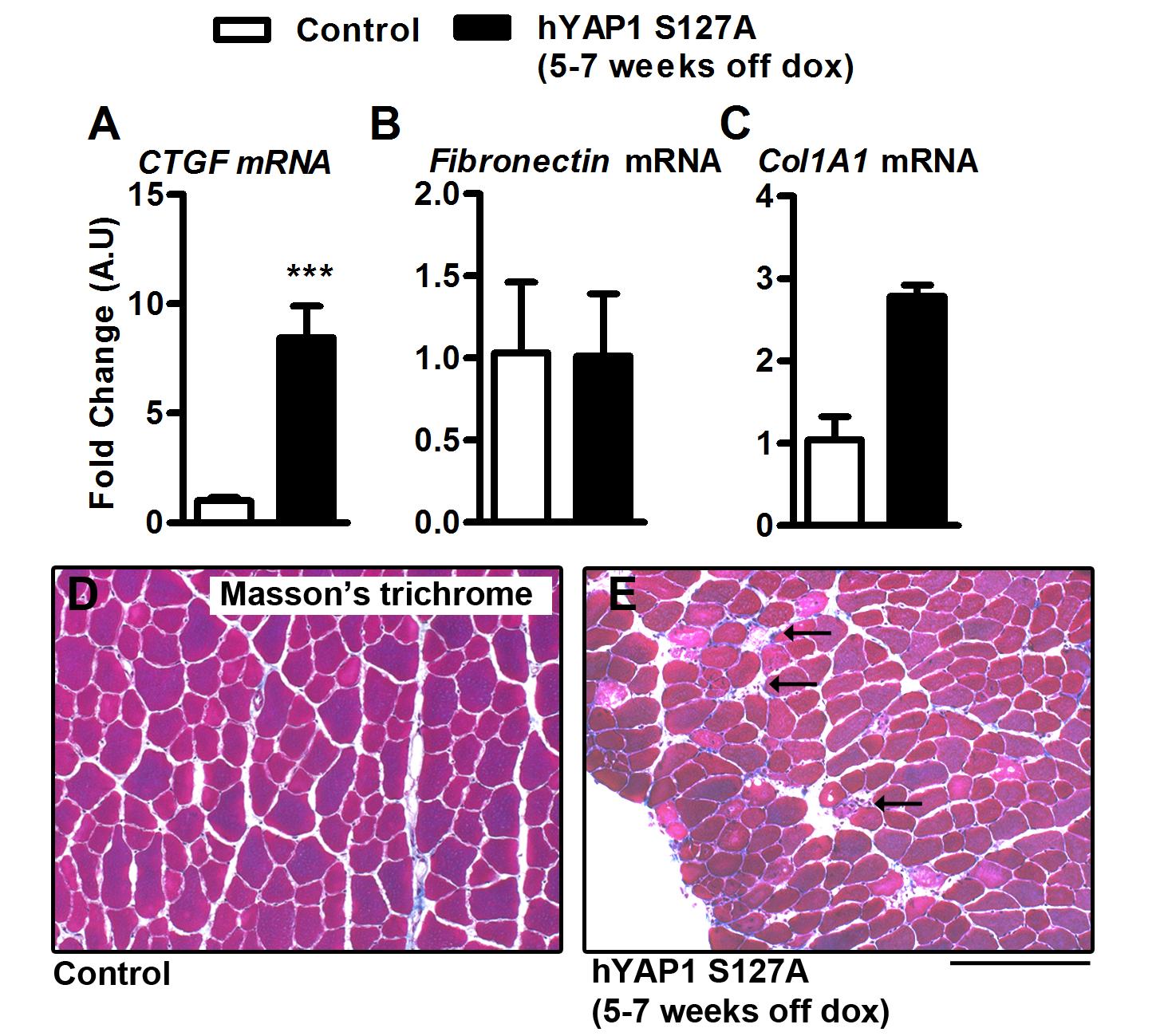

Supplement: Figure S3 — No significant fibrotic tissue accumulation in skeletal muscles of MCK-tTA-hYAP1 S127A mice. TA muscles of transgenic mice were harvested 5–7 weeks following doxycycline (dox) withdrawal. RNA was isolated and processed for qRT-PCR analysis of A) Contective tissue growth factor (Ctgf) mRNA, B) Fibronectin mRNA C) Type-1 Collagen (Col1A1) mRNA. D+E) Masson’s trichrome blue staining of TA sections from control and MCK-tTA-hYAP1 S127A mice. Black arrows highlight small areas of fibrotic tissue (blue staining). mRNA expression normalised to Gapdh mRNA. Values present mean ±SEM and displayed as fold change relative to control mice (n = 8). ***P<0.001. (TIF) [file pone.0059622.s003.tif]

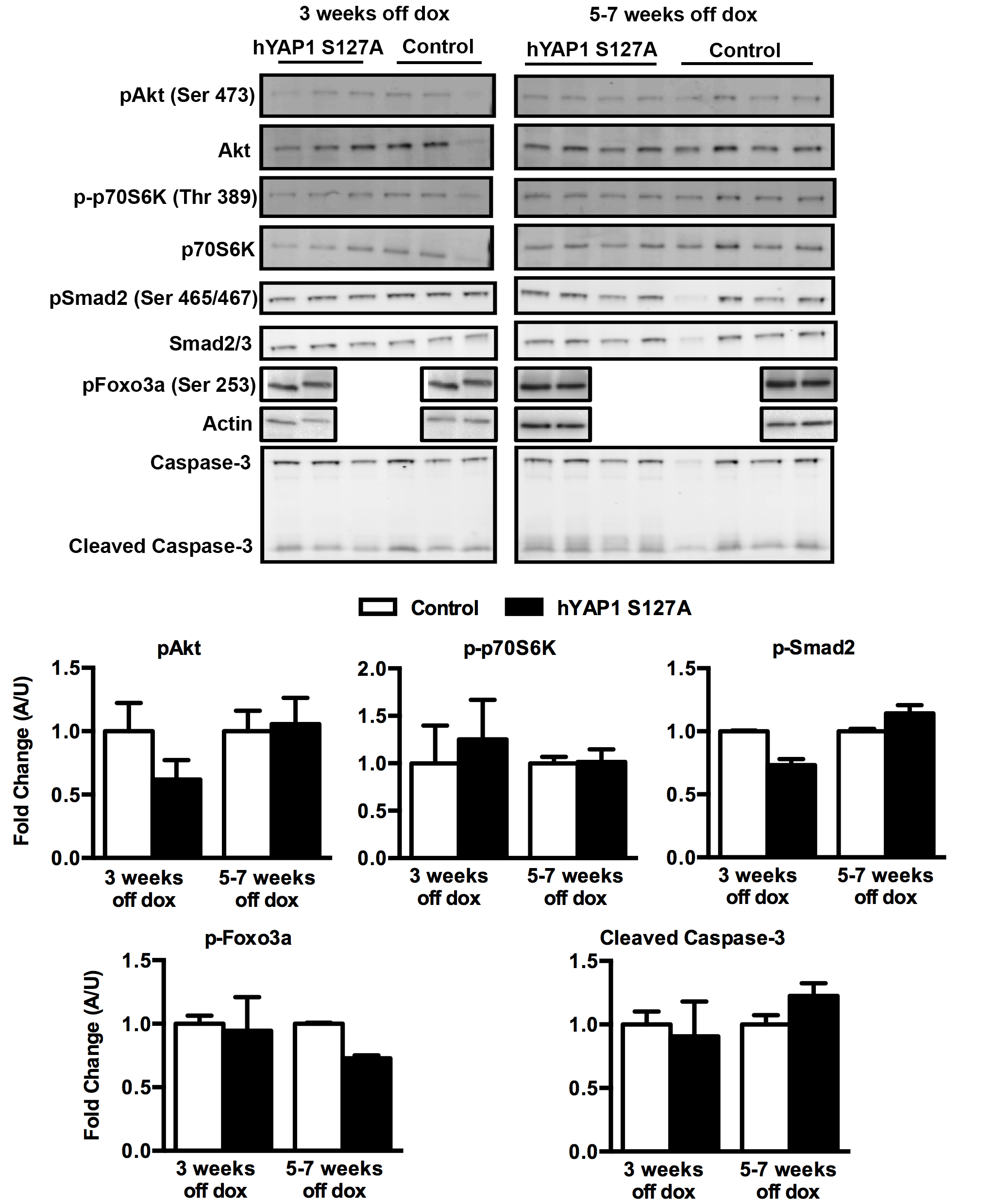

Supplement: Figure S4 — Cell signalling in skeletal muscle of MCK-tTA-hYAP1 S127A. TA muscles of transgenic mice were harvested 3 weeks or 5–7 weeks following doxycycline withdrawal. Protein was isolated from muscle samples and processed for Western blotting. Membranes were probed with indicated antibodies. Densitometry was performed and normalised to indicated total proteins for A) pAkt, B) p-p70S6K, C) pSmad, D) pFoxo3 and E) caspase. Values present mean ±SEM and displayed as fold change relative to control mice (n = 4–8). (TIF) [file pone.0059622.s004.tif]
