# Supplementary material for: Constitutive Expression of Yes-Associated Protein (Yap) in Adult Skeletal Muscle Fibres Induces Muscle Atrophy and Myopathy
Source: PLoS One. 2013 Mar 27;8(3):e59622. doi: 10.1371/journal.pone.0059622 (PMC3609830; doi:10.1371/journal.pone.0059622)
Supplement: Table S1 — Primer details. PCR primers for genotyping, end-point PCR primers for hYAP and Gapdh mRNA and qRT-PCR primers and probes (Roche Universal Probe Library). (DOCX) [file pone.0059622.s005.docx]

**Table S1**

PCR primers for genotyping

| Allele | Primer | Sequence |
| --- | --- | --- |
| MCK-tTA | Forward | 5'-CGCTGTGGGGCATTTTACTTTAG-3' |
|  | Reverse | 5'-CATGTCCAGATCGAAATCGTC-3' |
| TRE-hYAP1 S127A | Common (Forward) | 5'-CCCTCCATGTGTGACCAAGG-3' |
|  | Wildtype (Reverse) | 5'-GCACAGCATTGCGGACATGC-3' |
|  | Mutant (Reverse) | 5'-GCAGAAGCGCGGCCGTCTGG-3' |

MCK-tTA assay = 425 bp (mutant band)

TRE-hYAP1 S127A assay = 331 bp (wildtype band) and 551 bp (mutant band)

End-point PCR primers for *hYAP* and *Gapdh* mRNA

| Gene | Primer | Primer Sequence | Product Size |
| --- | --- | --- | --- |
| Gapdh | Forward | 5'- TGGTGAAGGTCGGTGTGAAC-3' | 581 bp |
|  | Reverse | 5'- CCATCACGCCACAGCTTTC-3' |  |
| hYAP1 | Forward | 5'-GACCCCCACTGGAGTAGTCT -3' | 270 bp |
|  | Reverse | 5’-TTCATGGCTGAAGCCGAGTT-3’ |  |

|  |
| --- |

qRT-PCR primers and probes (Roche Universal Probe Library)

| Gene | UPL Probe# | Primer | Primer Sequence |
| --- | --- | --- | --- |
| *Atrogin-1* | 53 | Forward | 5’-AGTGAGGACCGGCTACTGTG-3’ |
|  |  | Reverse | 5’-GATCAAACGCTTGCGAATCT-3’ |
| *Caspase-3* | 80 | Forward | 5’-GAGGCTGACTTCCTGTATGCTT-3’ |
|  |  | Reverse | 5’-AACCACGACCCGTCCTTT-3’ |
| *Ctgf* | 71 | Forward | 5’- TGACCTGGAGGAAAACATTAAGA-3’ |
|  |  | Reverse | 5’- AGCCCTGTATGTCTTCACACTG-3’ |
| *Col1A1* | 49 | Forward | 5’-TCCCCTGGAATCTGTGAATC-3’ |
|  |  | Reverse | 5’-TGAGTCGAATTGGGGAGAAT-3’ |
| *Embryonic Myosin* | 18 | Forward | 5’-GGATGGGAAAGTCACTGTGG -3’ |
|  |  | Reverse | 5’-GTCCTCTGGCTTAACCACCA-3’ |
| *Fibronectin* | 52 | Forward | 5’-CGGAGAGAGTGCCCCTACTA-3’ |
|  |  | Reverse | 5'-CGATATTGGTGAATCGCAGA-3' |
| *hYAP1* | 84 | Forward | 5'-CCCAGATGAACGTCACAGC-3' |
|  |  | Reverse | 5'-GATTCTCTGGTTCATGGCTGA-3' |
| *MuRF-1* | 17 | Forward | 5’-CCTGCAGAGTGACCAAGGA-3’ |
|  |  | Reverse | 5’-GGCGTAGAGGGTGTCAAACT-3’ |
| *Myf5* | 77 | Forward | 5'-CTGCTCTGAGCCCACCAG-3' |
|  |  | Reverse | 5'-GACAGGGCTGTTACATTCAGG-3' |
| *Myogenin* | 63 | Forward | 5'-CCTTGCTCAGCTCCCTCA-3' |
|  |  | Reverse | 5'-TGGGAGTTGCATTCACTGG-3' |
| *Pax7* | 85 | Forward | 5’-GGCACAGAGGACCAAGCTC-3’ |
|  |  | Reverse | 5’-GCACGCCGGTTACTGAAC-3’ |
